# Introduction and Outcomes From an Enhanced Physical Health Clinic for People With Intellectual Disabilities Prescribed Psychotropic Medication

**DOI:** 10.1192/bjo.2025.10524

**Published:** 2025-06-20

**Authors:** Indermeet Sawhney, Elizabeth Patteril, Regi Alexander, Mohamed Sathick, Tim Gale

**Affiliations:** Hertfordshire Partnership NHS Foundation Trust, Hertfordshire, United Kingdom

## Abstract

**Aims:** People with intellectual disabilities have higher rates of mental health difficulties than those without. The physical health inequalities and premature mortality that they experience is even more pronounced. In the United Kingdom, physical healthcare has traditionally been co-ordinated and delivered through primary healthcare settings. There is a case that physical health inequalities for those with intellectual disability and mental health difficulties can be reduced further if primary care interventions are supplemented by Enhanced Physical Health Clinics (EPHCs) co-located in mental health outpatient settings.This paper describes the structure and setting up of an EPHC for people with intellectual disability and mental disorders and an evaluation of its first 2 years.

**Methods:** The EPHC database which contains patient demographics and process data for the clinic regarding tests and interventions completed was utilised for this study. This includes sociodemographic, psychiatric, and physical health diagnoses, prescribed medication, physical health assessments and interventions.

**Results:** During its first two years, the clinic saw 463 patients. The mean age was 44 years and 62% were male. There was considerable developmental and psychiatric comorbidity, with high rates of autism and major mental illness. The most common physical health diagnoses were epilepsy, hypothyroidism, diabetes, hypertension, and asthma. A range of previously unidentified unmet healthcare needs that warrant further assessment and treatment was identified.

**Conclusion:** The EPHC was effective in promoting physical health monitoring and screening in a population which experiences significant health barriers. Recommendations regarding clinical practice and future research are provided.

